# Inhibition Effect and Mechanism Explanation of Perilla Seed Extract as a Green Corrosion Inhibitor on Q235 Carbon Steel

**DOI:** 10.3390/ma15155394

**Published:** 2022-08-05

**Authors:** Yu Li, Wenqiang Xu, Jiayu Lai, Sheng Qiang

**Affiliations:** 1 College of Water Conservancy and Hydropower, Hohai University, Nanjing 210098, China; 2 School of Civil Engineering and Architecture, Wuhan Institute of Technology, Wuhan 430079, China

**Keywords:** perilla seed extract, green corrosion inhibitor, luteolin, apigenin, chemical calculation

## Abstract

The development of environmentally friendly corrosion inhibitors has become a research hotspot. Aiming at the potential corrosion inhibition effect of perilla seed extract on Q235 carbon steel, the corrosion inhibition effect was quantitatively evaluated by various research methods, and the effective corrosion inhibition composition and mechanism were discussed. The research methods include potentiodynamic polarization curve method, HPLC-MS, FT-IR, XPS and chemical calculation. The experimental results show that the inhibitor prepared from perilla seed extract is a mixed inhibitor, and its adsorption behavior accords with Langmuir adsorption theory and its adsorption free energy is −22.70 kJ/mol. Combined with the experimental results and theoretical calculation, the effective corrosion inhibiting components are luteolin and apigenin. Theoretical calculation shows that both of them are adsorbed parallel to the surface of carbon steel to form thin films. The adsorption mechanism is that carbonyl O atoms in luteolin and apigenin hybridize with the 3 d empty orbit of Fe. From the point of view of quantum chemistry, the smaller the HOMO value and the energy gap value, the better the adsorption of corrosion inhibitor on the surface of carbon steel. From the point of view of molecular dynamics simulation, the greater the absolute value of adsorption energy, the better the adsorption of corrosion inhibitor on carbon steel surface.

## 1. Introduction

At present, reinforced concrete and steel fiber concrete have been widely used in the construction of various coastal infrastructures, such as sea-crossing bridges, subsea tunnels, underground rail transit, ports and so on [1,2]. These infrastructures are exposed to the penetration and diffusion of corrosive ions in seawater or groundwater during service [3,4]. As a result, the passive film of the steel bars or steel fibers in the concrete is locally damaged, and various iron oxides are generated, which can cause local corrosion and expansion, destroy the bonding of the concrete structure, reduce the service life or cause the safety failure of the building structure [5].

Corrosion inhibitors are widely used as an economical and effective means of anti-corrosion [6]. The main function of corrosion inhibitors is to inhibit the bipolar reaction of electrochemical corrosion. The specific method is that the corrosion inhibitor will react on the metal surface to form an adsorption film, thereby increasing the corrosion potential or increasing the charge transfer resistance [7], so as to achieve the effect of inhibiting or slowing down the corrosion. Therefore, a certain amount of corrosion inhibitor is often added in engineering to protect or delay the corrosion of steel bars [8].

Corrosion inhibitors can be divided into anodic, cathodic and composite types according to their mechanism of action [9]. Anodic corrosion inhibitors represented by inorganic salts such as chromate, molybdate and nitrite mainly inhibit metal corrosion by preventing or slowing down the electron loss process of the anode [10]. On the contrary, cathodic corrosion inhibitors inhibit corrosion by changing the electron-acquiring ability of the cathode, typically represented by phosphates, zincates and higher fatty acid ammonium salts [11]. The composite corrosion inhibitor has both the advantages of the above two corrosion inhibitors. This kind of corrosion inhibitor is often obtained by reasonable collocation and experimental verification of several oxidative anodic corrosion inhibitor components and cathodic corrosion inhibitor components that can generate insoluble salts [12].

In terms of chemical composition, corrosion inhibitors are mainly divided into inorganic type, organic type and composite type. The inorganic acid ions in the inorganic corrosion inhibitor and Cl^−^ form a competitive adsorption on the metal surface [13], forming a passivation film. However, the inorganic acid ion is unstable, and its content will continue to decrease during long-term service, and the local mineral acid ion concentration will be insufficient on the metal surface [14]. At this time, the inorganic acid ions will act as an oxidant to promote the dissolution of the passive film on the metal surface and accelerate the corrosion of the metal. At the same time, some inorganic acid salts contain high-valence heavy metal ions, which are toxic to a certain extent and cause certain damage to the environment and health, so their use is also restricted to a certain extent. The corrosion inhibition mechanism of organic corrosion inhibitors is mainly that functional groups containing heteroatoms such as N, O, S and other structures in organic molecules and structures such as benzene rings are adsorbed on the metal surface [15] to form an adsorption film. The corrosion inhibition efficiency has a certain relationship with the molecular configuration of the corrosion inhibitor, the hydrophilicity and hydrophobicity of the molecule and the compatibility with the metal interface. However, at present, such organic corrosion inhibitors are mainly obtained through chemical production or are obtained from by-products in the chemical production process, the production process is complicated and the economic and ecological costs are high.

At present, researchers have begun to develop green and environmentally friendly corrosion inhibitors, among which corrosion inhibitors based on plant extracts [16,17] have great potential application value. The plants with certain corrosion inhibition effect reported by academic circles include poppy [17], garlic [18], pepper [19], tea [20], orange peel [21], ragweed [22], mint [23], etc. There are still some deficiencies in the research on green and environmentally friendly plant extract corrosion inhibitors. Most studies pay less attention to the protection of green corrosion inhibitors against metal corrosion in alkaline environments, and only a few studies can clearly point out the effective corrosion inhibitor components, molecular structures and corrosion inhibition mechanisms in plant extract corrosion inhibitors.

In this paper, the corrosion inhibition effect of perilla seed extract on Q235 carbon steel in simulated pore liquid of concrete was studied. Q235 carbon steel is an alloy with iron and carbon as main components. It is widely used in ordinary concrete structures, formwork and unimportant steel frame structures. By setting different inhibitor concentrations, the potentiodynamic polarization curve method was used to quantitatively evaluate the corrosion inhibition efficiency. Combined with micro-characterization methods such as HPLC-MS, ATR-FTIR and XPS, the effective corrosion inhibitor components were determined. Through theoretical calculation, the adsorption characteristics and microscopic mechanism of corrosion inhibitor components were found.

## 2. Materials and Methods

### 2.1. Chemicals and Reagents

The chemicals and reagents used in this paper were purchased from Tianjin Zhiyuan Chemical Reagent Co., Ltd. (Tianjin, China) Potassium hydroxide solution is a prepared 0.6 mol/L standard solution. The specifications of sodium hydroxide, calcium hydroxide, sodium chloride and absolute ethanol are analytically pure (≥96.5%), analytically pure (≥95.0%), analytically pure (≥99.0%) and analytically pure (≥99.0%), respectively.

### 2.2. Preparation of Corrosion Inhibitor Solution and Simulated Concrete Pore Solution

The simulated concrete pore solution adopts the ratio of 0.6 mol/L KOH, 0.2 mol/L NaOH and saturated Ca(OH)_2_, and 3% NaCl is added to the simulated pore liquid.

The perilla seed used in this paper is produced in Bozhou, Anhui Province, China. In this paper, the corrosion inhibitor solution is prepared by grinding mature and dried perilla seeds into powder, and then passing through a 40-mesh sieve. Accurately weigh a certain mass of perilla seed powder, dissolve it in a certain volume of concrete simulated pore fluid. Concentrations of corrosion inhibitors were 0 g/L, 1 g/L, 2 g/L, 3 g/L and 4 g/L, respectively. Soak for 24 h and shake the solution with ultrasonic wave for 10 min every 4 h. After the preparation is completed, leave the carbon steel electrode pre-film for 2 h. The definition of the corrosion inhibitor concentration is as follows:

(1) $$C=M_{\mathrm{perilla}\;\mathrm{seed}}/V$$

In the formula, C is the concentration of corrosion inhibitor in g/mL, $M_{\mathrm{perilla}\;\mathrm{seed}}$ is the mass of perilla seed powder accurately weighed in g and V is the volume of simulation concrete pore solution in L.

### 2.3. Potentiodynamic Polarization Curve Test

In the test system, the auxiliary electrode is a Pt electrode, and the reference electrode is an Ag/AgCl (saturated KCl solution) electrode. During the test, the carbon steel electrode was taken out from the corrosion inhibitor solution and placed in a three-electrode system of concrete-simulating pore solution, and the test was carried out after 30 min. The polarization range was around −1200 mV to + 200 mV, the sampling frequency was 5 Hz and the scan rate was 5 mV/s. By fitting the polarization curve, the corrosion potential E_corr_ and the self-corrosion current density I_corr_ are obtained. The corresponding corrosion inhibition efficiency can be calculated by the following formula:

(2) $$IE\%=\frac{i_{0}-i}{i_{0}}\times 100$$

In the formula, IE% is the corrosion inhibition efficiency and $i_{0}$ and i are the corrosion currents of the carbon steel electrode without and with corrosion inhibition treatment, respectively, in A/cm^2^.

### 2.4. Detection and Characterization Methods of Corrosion Inhibition Effect

#### 2.4.1. HPLC-MS Test

The main chemical components in perilla seed extract were confirmed by HPLC-MS method and the instrument model for chromatographic detection was Waters 2695. Liquid chromatography conditions: C18 liquid chromatography column (250 mm × 4.6 mm i.d., 3 μm), column temperature is 30 °C. Pass the sample through a 0.22 μm filter membrane with a flow rate of 1 mL/min and an injection volume of 10 μL. Mobile phase: A is KH_2_PO_3_ ultrapure aqueous solution with pH = 2, B is acetonitrile and A:B = 95:5, gradient running time is 20 min. The detection wavelength is 280 nm. The instrument model for mass spectrometry detection is Waters ZQ2000, which uses electrospray ion source and negative ion mode scanning. The ion source temperature is 140 °C, the atomization temperature is 350 °C and the voltage is 3.5 kV.

#### 2.4.2. ATR-FTIR Test

The FT-IR test can obtain the information of the corrosion inhibitor solution and the functional group on the surface of the working electrode. Therefore, the FT-IR test was carried out on the filtrate of perilla seed powder extracted with absolute ethanol and the surface of the working electrode pre-filmed in the corrosion inhibitor solution. The working electrode surface needs to be dried for 24 h before testing. The model of the testing instrument is Thermo Scientific Nicolet iS5 (Waltham, MA, USA), the test mode is ATR mode, the spectral resolution is 4 cm^−1^, the number of scans is 32 and the wave number range is 600–4000 cm^−1^.

#### 2.4.3. XPS Test

XPS test can obtain information such as elements and chemical bonds on the electrode surface. Therefore, the XPS test was performed on the surface of the working electrode and the model of the testing instrument was Thermo Scientific K-Alpha. The excitation source is Al Kα rays (hv = 1486.6 eV), the pass energy of the full spectrum scan is 100 eV and the step length is 1 eV; the pass energy of the fine spectrum scan is 50 eV and the step length is 0.1 eV. The obtained XPS spectra were calibrated for the C_1s_ peak (284.8 eV), and the fine spectra of each element were peak-fitted. The peak-fitting mainly refers to the XPS standard spectra and similar literature.

### 2.5. Chemistry Calculation

#### 2.5.1. Quantum Chemical Calculation

With the help of microscopic characterization, the effective corrosion inhibitor in perilla seed was determined, the molecular model was established, the geometric structure was optimized and the single point energy was calculated. The energy values of the highest occupied orbital (HOMO), the lowest unoccupied orbital (LUMO) and the energy gap ΔE (|E_HOMO_−E_LUMO_|) of the effective corrosion inhibitor were obtained. Global parameters such as chemical potential, hardness, softness, electrophilic index and local parameters such as Fukui function are obtained based on the calculation results of frontier orbital distribution, which can be used to analyze the local reactivity and adsorption active sites of corrosion inhibitors.

In this paper, the generalized gradient approximation (GGA)-PW91 exchange correlation function of density functional theory (DFT) is used, and the basis set is set as double numerical basis set (DNP) expanded by polarization function, so as to optimize the geometric configuration of corrosion inhibitor molecules. Then, the frontier orbit distribution and local parameters are calculated at the level of DNP basis set.

The convergence criteria of geometry optimization and energy calculation are set as follows: the energy tolerance of SCF of each atom is 1 × 10^−5^ Ha, the maximum force tolerance is 2 × 10^−3^ eV·Å^−1^ and the maximum displacement tolerance is 5 × 10^−3^ Å. The maximum SCF cycle is set to 500 cycles and the maximum iteration step is 0.05 Å.

#### 2.5.2. Molecular Dynamics Simulation

Molecular dynamics simulation can give the adsorption position and adsorption energy, and further describes the interface interaction between the corrosion inhibitor and the carbon steel substrate. Two possible substrates, the Fe substrate and γ-FeOOH substrate, are considered in this paper. Metals such as steel bars or steel fibers will form a passivation film dominated by γ-FeOOH in the alkaline environment of the concrete pore liquid, and the Fe matrix will be only exposed in a few surface defects. However, such places are often the most vulnerable to attack by aggressive ions, forming pitting pits. Whether the corrosion inhibitor molecules can be adsorbed on the Fe matrix is the key to preventing pitting corrosion, and the corrosion inhibitor molecules adsorbed on the γ-FeOOH substrate can form a more complete adsorption film to further protect the internal Fe matrix. Therefore, it is necessary to study both the Fe substrate and γ-FeOOH substrate.

**Figure 1:**
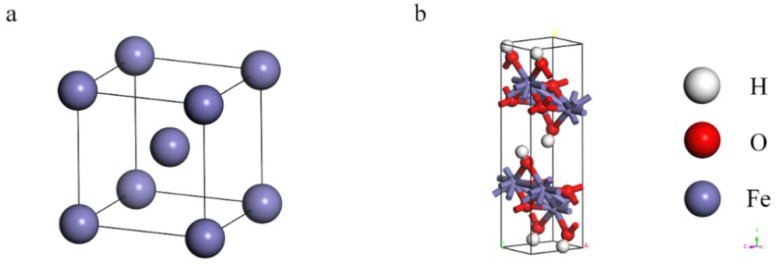
Crystal structures: (**a**) Fe and (**b**) γ-FeOOH.

**Figure 2:**
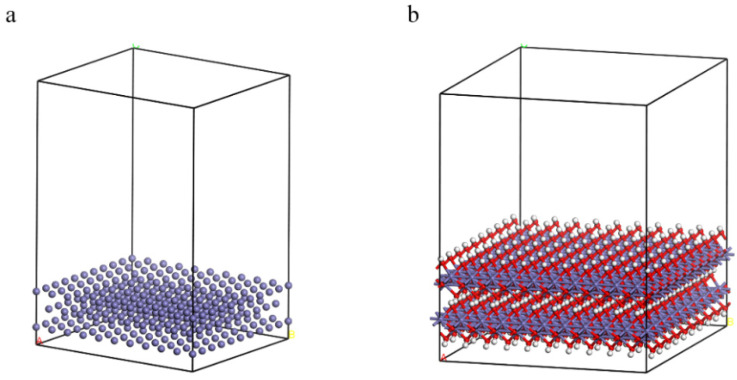
Interface models: (**a**) Fe and (**b**) γ-FeOOH.

Figure 1 presents the crystal structures of Fe and γ-FeOOH. Fe belongs to the cubic crystal system, the space point group is IM-3M, the lattice parameters are a = b = c = 2.87 Å, α = β = γ = 90°. γ-FeOOH belongs to the orthorhombic crystal system, the space point group is CMC21, and the lattice parameters are a = 3.07 Å, b = 12.53 Å, c = 3.88 Å, α = β = γ = 90°. In the process of model building, two stable surfaces with the lowest Miller indices of crystal structures, namely, Fe (110) surface and γ-FeOOH (010) surface, were selected as exposed surfaces. Figure 2 presents the interface model of Fe and γ-FeOOH. The interface model of Fe (110) consists of 3 layers with a total of 300 Fe atoms, and the model size is 24.82 Å × 24.82 Å × 30.00 Å. The interface model of γ-FeOOH (010) consists of 8 layers with a total of 400 Fe atoms, 800 O atoms and 400 H atoms, and the model size is 38.76 Å × 30.70 Å × 38.59 Å. To improve computational efficiency, all the base atoms of the interface model are frozen.

The interface interaction between the effective corrosion inhibitor of perilla seed and the surface of carbon steel was studied by MD simulation. MD simulation adopts NVT ensemble, uses COMPASSIII force field, temperature is 298 K, total simulation time is 500 ps and time step is 1 fs. The adsorption energy ($E_{adsorption}$) of effective corrosion inhibitors on the surface of carbon steel is calculated by the following formula:

(3) $$E_{adsorption}=E_{total}-E_{metal}-n\cdot E_{inhibitor}$$

Here, $E_{total}$ is the total energy of the system, $E_{metal}$ is the energy of carbon steel substrate and $n\cdot E_{inhibitor}$ is the energy of n corrosion inhibitor molecules.

## 3. Results and Discussion

### 3.1. Evaluation of Corrosion Inhibition Efficiency

#### 3.1.1. Influence of Corrosion Inhibitor Concentration on Corrosion Inhibition Efficiency

**Figure 3:**
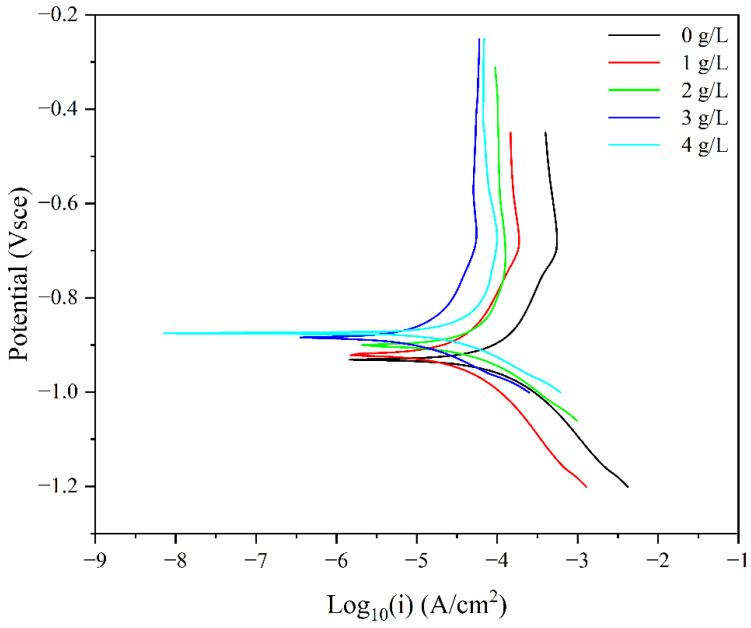
Potentiodynamic polarization curves of carbon steel electrodes in different concentrations of corrosion inhibitor solutions.

Figure 3 shows the potentiodynamic polarization curves of carbon steel electrodes in different concentrations of corrosion inhibitor solutions. Observing Figure 3, the overall form of the polarization curve has not changed, indicating that the corrosion inhibitor does not change the basic properties of the carbon steel surface. However, as a whole, as the concentration of the corrosion inhibitor increases, the polarization curve shifts to the left and up, which indicates that the corrosion inhibitor prepared by perilla seed extract effectively reduces the corrosion current and increases the corrosion potential.

According to the polarization curve in Figure 3, the corrosion potential and self-corrosion current are obtained by extrapolation method, as shown in Table 1, where the corrosion inhibition efficiency IE% is calculated by Equation (2). It can be seen from Table 1 that with the gradual increase in the corrosion inhibitor concentration in the simulated concrete pore solution, the corrosion potential of the carbon steel electrode moves in the positive direction, from −932 mV to −875 mV. At the same time, the corrosion current is constantly decreasing, from 1.11 × 10^−4^ A/cm^2^ to 1.18 × 10^−5^ A/cm^2^, and the corrosion inhibition efficiency is also increased from 65.41% of 1 g/L to 89.39% of 4 g/L.

From the above results, it can be found that the corrosion inhibitor prepared by perilla seed extract is a mixed-type corrosion inhibitor, which can inhibit the corrosion of the anode and the cathode at the same time.

#### 3.1.2. Adsorption Behavior of Perilla Seed Corrosion Inhibitor on Carbon Steel Surface

Three classical adsorption theories are used to analyze the adsorption characteristics of the corrosion inhibitor on the surface of carbon steel [24,25,26]. The expressions are as follows:

(4) $$Langmuir:\;\frac{C}{\theta }=\frac{1}{K_{ads}}+C$$

(5) $$Temkin:\;\exp\left(-2a\theta \right)=K_{ads}C$$

(6) $$Frumkin:\;\frac{\theta }{1-\theta }\exp\left(-2a\theta \right)=K_{ads}C$$

In the formula, C is the concentration of corrosion inhibitor in g/L. θ is the coverage area ratio of the corrosion inhibitor. According to the similar research of K. Shalabi [27] and M. Messali [28], θ can be replaced by the corrosion inhibition efficiency IE% in Table 1 obtained by potentiodynamic polarization test. $K_{ads}$ is the adsorption constant in L/g. a represents the interaction between corrosion inhibitor molecules.

Meanwhile, the adsorption free energy $\Delta G_{ads}$ of the corrosion inhibitor can be calculated by the following formula:

(7) $$\Delta G_{ads}=-RTln\left(1\times 10^{3}K_{ads}\right)$$

In the formula, R is the ideal gas constant, its value is 8.314 J/mol/K. T is the absolute temperature and 285 K. 1 × 10^3^ is the concentration of water molecules in g/L.

**Figure 4:**
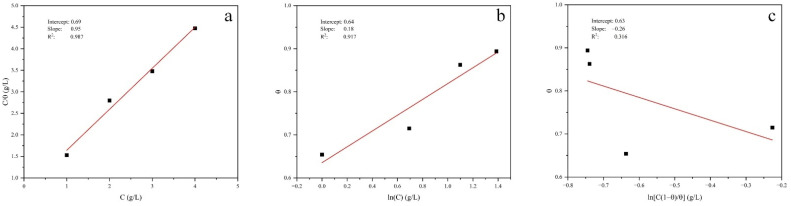
Fitting results of three adsorption theories: (**a**) Langmuir, (**b**) Temkin and (**c**) Frumkin.

Figure 4 is a fitting result of three adsorption theories. According to the goodness of fit, the adsorption behavior of the green corrosion inhibitor, which prepared from perilla seed extract on the surface of carbon steel, conforms to the Langmuir adsorption theory. The corresponding free energy of adsorption is −22.70 kJ/mol. According to the similar research of M. Messali [28], the free energy greater than −20 kJ/mol is attributed to the physical adsorption between inhibitor molecules and metal surface, while the free energy less than −40 kJ/mol involves the charge sharing or transfer between inhibitor molecules and metal surface, which belongs to chemical adsorption. The free energy is between the two, which indicates that the adsorption mechanism of corrosion inhibitor molecules on metal surface combines chemical adsorption and physical adsorption. Therefore, the corrosion inhibitor developed in this paper belongs to the complex mechanism of chemical adsorption and physical adsorption.

### 3.2. Determination of Chemical Constituents in Perilla Seed Extract

**Figure 5:**
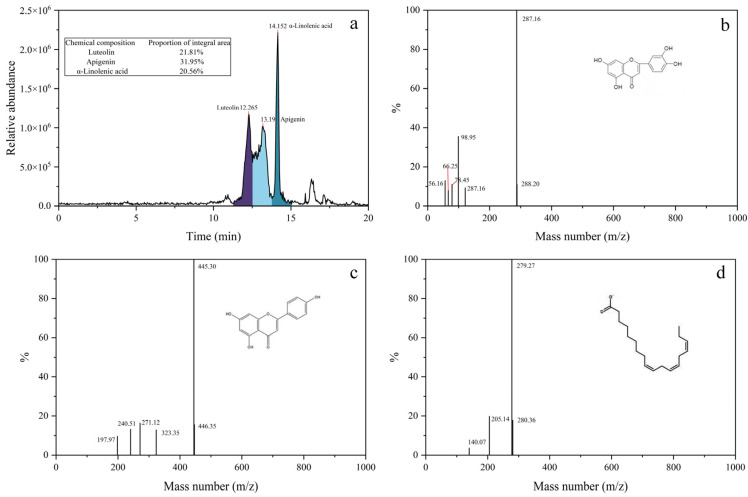
Perilla seed extract’s (**a**) chromatography and mass spectrometry: (**b**) luteolin, (**c**) apigenin and (**d**) α-linolenic acid.

After the potentiodynamic polarization test, it can be confirmed that the perilla seed extract contains mixed corrosion inhibitors that can inhibit the corrosion of Q235 carbon steel. In order to further explore the effective ingredients, this paper uses HPLC-MS combined detection methods to obtain the main chemical components in perilla seeds. As shown in Figure 5a, perilla seeds mainly contain three organic substances: luteolin, apigenin and α-linolenic acid, the contents of which are 21.81%, 31.95% and 20.56%, respectively. The three organics can be confirmed from the strong peaks on the rightmost side of the corresponding mass spectra in Figure 5b–d. These three strong peaks correspond to the molecular ion peaks of luteolin, apigenin and α-linolenic acid, respectively.

### 3.3. Changes in Functional Groups in the Surface Film

**Figure 6:**
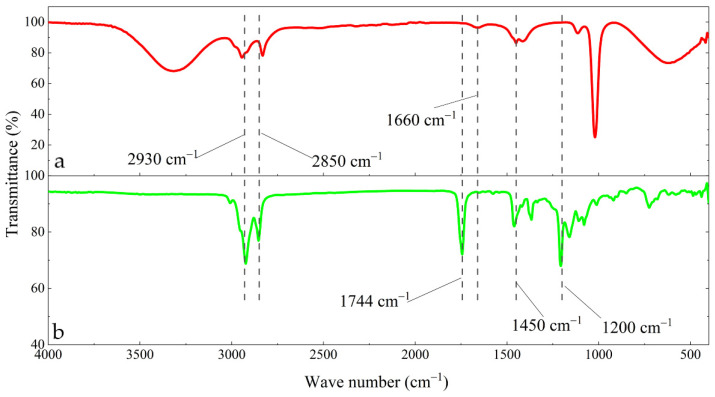
The spectrums of (**a**) the corrosion inhibitor solution (4 g/L) and (**b**) the surface of the carbon steel electrode in the corrosion inhibitor solution (4 g/L).

Figure 6 shows the FT-IR comparison between the corrosion inhibitor solution prepared by perilla seed extract and the surface of the carbon steel electrode. The position of each characteristic peak is marked in the figure. In total, 2930 cm^−1^ and 2850 cm^−1^ are the asymmetric stretching vibration and symmetric stretching vibration of the C-H bond, respectively [29]. Overall, 1744 cm^−1^ is the stretching vibration of the carbonyl group [30] and 1660 cm^−1^ is the stretching vibration of the C=C bond [31]. The 1450 cm^−1^ and the weak peak to the left are the stretching vibration of the benzene structure skeleton [32] and 1200 cm^−1^ is the stretching vibration of the phenolic hydroxyl group [33]. On the whole, the main characteristic peaks on the electrode surface are similar to the main characteristic peaks of the corrosion inhibitor solution, which once again shows that the effective components in the corrosion inhibitor are adsorbed on the surface of the carbon steel electrode to form a surface film, which has the effect of inhibiting electrode corrosion. At the same time, each main characteristic peak also corresponds to the functional groups of luteolin, apigenin and α-linolenic acid. Therefore, through this test, it can be basically confirmed that the effective corrosion inhibitors in perilla seeds are luteolin, apigenin and α-linolenic acid. The difference is that there are obvious carbonyl groups and phenolic hydroxyl groups on the surface of the carbon steel electrode pre-filmed in the corrosion inhibitor solution, and the presence of the halogen ion Cl^−^ causes the peak position of the carbonyl group to shift to a high wave number. At this time, luteolin and apigenin in the corrosion inhibitor solution are mainly adsorbed on the surface of carbon steel to form a film.

### 3.4. Effect of Effective Anti-Corrosion Ingredients on the Surface Film

**Figure 7:**
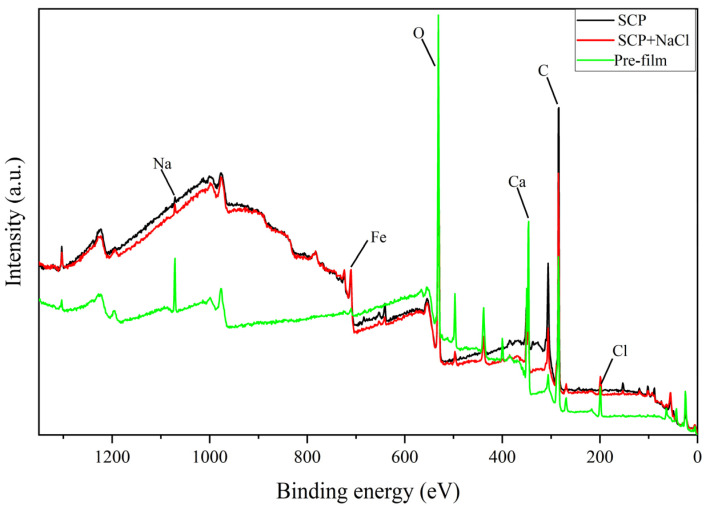
XPS full spectrums of carbon steel electrode surface in different environments.

After confirming that the perilla seed extract has corrosion inhibition effect and effective corrosion inhibition components, XPS method was used to further study the effect of effective corrosion inhibition components on the surface composition and chemical valence state of carbon steel electrodes. Figure 7 shows the full spectra of three electrode surfaces under different conditions. The conditions are simulated concrete pore solution (SCP), simulated concrete pore solution with NaCl (SCP + NaCl) and simulated concrete pore solution after being placed in corrosion inhibitor for two hours (pre-film). It can be found from the figure that C and O elements are obviously present on the surfaces of the three electrodes. For Na and Cl, these two elements are almost absent on the electrode surface of SCP. However, the Cl element under the other two conditions is not very different, but the electrode surface with the corrosion inhibitor pre-film has obvious adsorption to Na and Ca, which is related to the strong electronegativity of the corrosion inhibitor.

**Figure 8:**
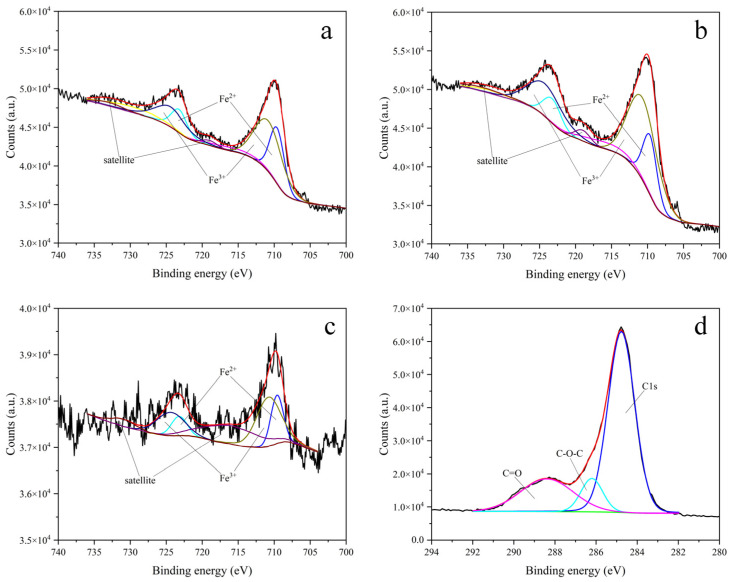
Fine spectra of Fe on the surface of carbon steel electrodes in different environments. (**a**) Simulated concrete pore solution, (**b**) simulated concrete pore solution mixed with 3% NaCl, (**c**) corrosion inhibitor solution and C on the surface of carbon steel electrodes in different environments. (**d**) Corrosion inhibitor solution.

Figure 8a–c show the fine spectrums and peak fitting of Fe element under three different conditions. Comparing Figure 8a,b, the electrode surfaces in SCP and SCP + NaCl have two valence states of Fe^2+^ (709.6 eV [34]) and Fe^3+^ (710.8 eV [34]). However, due to the presence of Cl^−^, the relative content of Fe^3+^ on the electrode surface increases, which indicates that the presence of Cl^−^ destroys the oxide film formed on the electrode surface in an alkaline environment and accelerates the corrosion of the electrode surface. Observing Figure 8c, the relative content of Fe^3+^ on the electrode surface was significantly reduced after adding the perilla seed corrosion inhibitor and pre-filming.

Figure 8d presents the fine spectrum and sub-peak fitting of element C. Observing Figure 8d, after adding the perilla seed corrosion inhibitor and pre-filming, C1s (284.8 eV [35]), C-O-C (286.5 eV [36]) and C=O (288.3 eV [37]) appeared on the electrode surface. This indicated that luteolin and apigenin were adsorbed on the carbon steel surface.

### 3.5. Analysis of Quantum Chemistry Calculation

#### 3.5.1. Frontier Orbit Distribution

Green corrosion inhibitor prepared from perilla seed extract belongs to organic corrosion inhibitor, and its corrosion inhibition effect is closely related to molecular structure and electronic structure [38]. At present, quantum chemical calculation is often used to study the structure–activity relationship of organic corrosion inhibitors. According to the frontier orbital theory [39], the distribution of the highest and lowest occupied orbitals of organic matter and the relative size of energy gap determine whether organic matter can undergo adsorption reaction.

**Figure 9:**
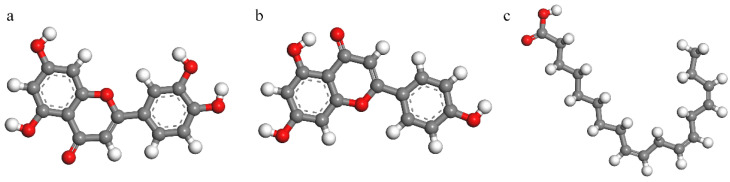
The optimized geometric configurations: (**a**) luteolin, (**b**) apigenin and (**c**) α-linolenic acid.

**Figure 10:**
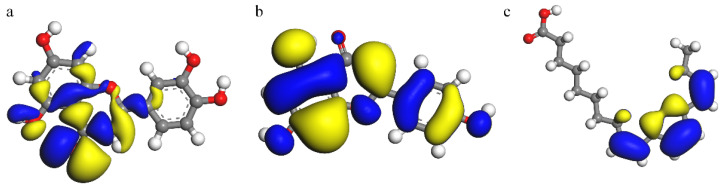
HOMO: (**a**) luteolin, (**b**) apigenin and (**c**) α-linolenic acid.

**Figure 11:**
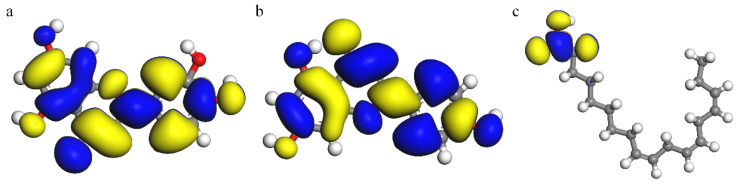
LUMO: (**a**) luteolin, (**b**) apigenin and (**c**) α-linolenic acid.

Figure 9, Figure 10 and Figure 11 show the molecular structure, HOMO and LUMO distribution of luteolin, apigenin and α-linolenic acid, respectively. Looking at Figure 10a,b and Figure 11a,b, it can be found that the orbital distributions of luteolin and apigenin are similar. The charge density of HOMO is mainly delocalized around the O atom of carbonyl group, and that of LUMO is mainly delocalized around the unsaturated bond of C ring of flavonoids. Looking at Figure 10c and Figure 11c, it can be found that the charge density of HOMO in α-linolenic acid is mainly delocalized around unsaturated double bonds, and that of LUMO is mainly delocalized in carboxylic acid groups.

Table 2 shows the HOMO, LUMO and energy gap values of luteolin, apigenin and α-linolenic acid. The charge density of HOMO and LUMO of luteolin and apigenin is similar, and the energy gap values are 2.571 eV and 2.650 eV, respectively. Compared with α-linolenic acid, the HOMO charge density of luteolin and apigenin is smaller, which means that their electron-donating ability is stronger. The energy gap is lower than that of α-linolenic acid (4.447 eV), the molecular stability is worse, and it is easier to participate in the adsorption reaction on the electrode surface.

#### 3.5.2. Local Reactivity

**Figure 12:**
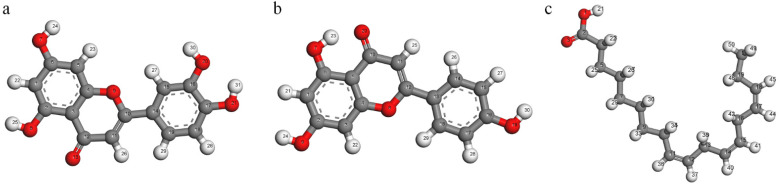
The atomic coding: (**a**) luteolin, (**b**) apigenin and (**c**) α-linolenic acid.

Figure 12 shows the atomic codes of luteolin, apigenin and α-linolenic acid, which correspond to the atomic codes of Fukui functions recorded in Table 3, Table 4 and Table 5. By studying the frontier orbital distribution of three corrosion inhibitors, the possible adsorption active sites were preliminarily determined. In order to further determine the specific adsorption active sites, the Fukui function of local reactivity should be used for analysis. Table 3, Table 4 and Table 5 show the Fukui functions of three corrosion inhibitor molecules, in which the larger the fi^+^ is, the more the atom is the nucleophilic reaction center, and the larger the fi^−^ is, the more the atom is the electrophilic reaction center. It is found that the distribution of nucleophilic/electrophilic reaction centers is basically the same as that of the frontier orbit. By analyzing the oxygen coding of nucleophilic/electrophilic reaction center, it is found that the nucleophilic/electrophilic ability of carbonyl oxygen of luteolin and apigenin is the strongest, and that of double bond oxygen of α-linolenic acid is the strongest.

### 3.6. Analysis of Molecular Dynamics Simulation

**Figure 13:**
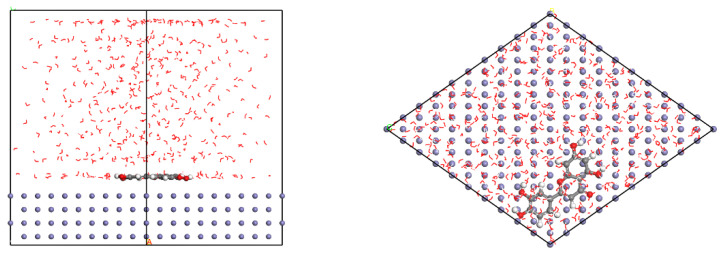
The adsorption configuration of luteolin molecule on Fe (110) surface.

**Figure 14:**
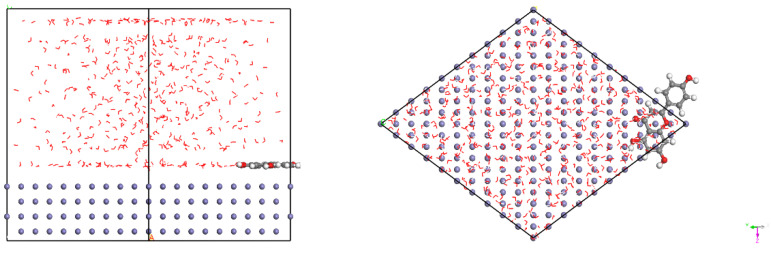
The adsorption configuration of apigenin molecule on Fe (110) surface.

**Figure 15:**
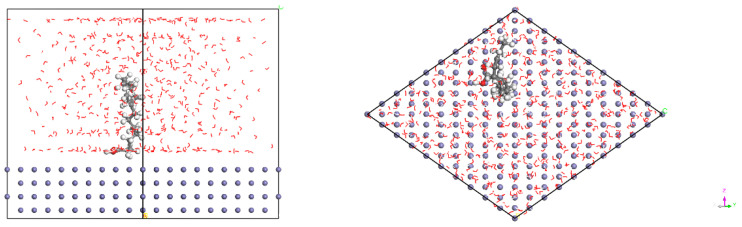
The adsorption configuration of α-linolenic acid molecule on Fe (110) surface.

**Figure 16:**
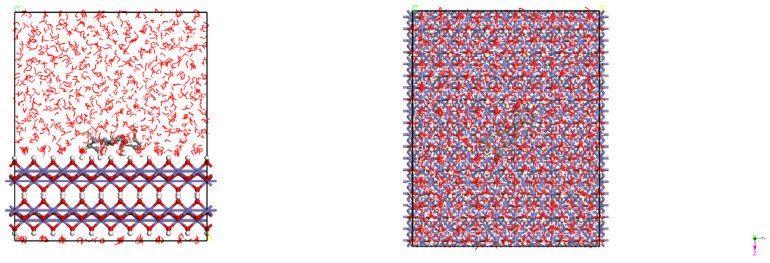
The adsorption configuration of luteolin molecule on γ-FeOOH (010) surface.

**Figure 17:**
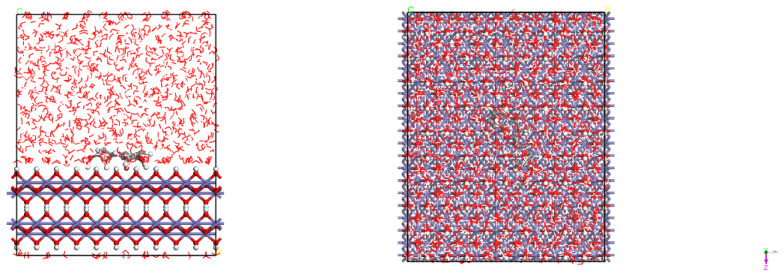
The adsorption configuration of apigenin molecule on γ-FeOOH (010) surface.

**Figure 18:**
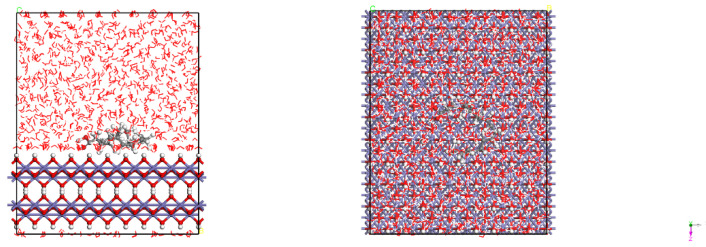
The adsorption configuration of α-linolenic acid molecule on γ-FeOOH (010) surface.

Figure 13 and Figure 14 show the adsorption configurations of luteolin and apigenin on Fe (110) surface in equilibrium, and the two organic molecules are parallel to the substrate surface. Figure 15 shows the adsorption configuration of α-linolenic acid on the surface of Fe (110) in equilibrium. The carboxylic acid group of α-linolenic acid is adsorbed on the surface of the substrate, keeping a vertical relationship with the surface. Figure 16 and Figure 17 show the adsorption configurations of luteolin and apigenin on γ-FeOOH (010) surface equilibrium, and the two organic molecules are still parallel to the substrate surface. Figure 18 shows the adsorption configuration of α-linolenic acid when γ-FeOOH (010) surface is in equilibrium. The carboxylic acid groups of α-linolenic acid are also adsorbed on the substrate surface in parallel.

According to the adsorption energies in Table 6, the adsorption energies of three organic molecules on the surface of Fe (110) and γ-FeOOH (010) are all negative, indicating that the adsorption is spontaneous, but the adsorption energies of inhibitor molecules on γ-FeOOH (010) are obviously smaller. On the surface of Fe (110), the adsorption energies of luteolin and apigenin are similar, −144.175 kJ/mol and −141.949 kJ/mol, respectively. The adsorption energy of α-linolenic acid is smaller than the former two, only −133.046 kJ/mol. This is consistent with the law of energy gap value. Combined with the above-mentioned FT-IR and XPS results, the adsorption film mainly composed of luteolin and apigenin was formed on the surface of carbon steel electrode pre-coated in the corrosion inhibitor solution prepared in this paper. The adsorption mechanism is that carbonyl O atoms in luteolin and apigenin hybridize with the 3 d empty orbit of Fe.

## 4. Conclusions

According to the potential corrosion inhibition effect of perilla seed extract, this paper obtained the following conclusions by means of potentiodynamic polarization curve method, HPLC-MS, FT-IR, XPS and computer technology such as quantum chemical calculation and molecular dynamics simulation:

(1) The corrosion inhibitor solution prepared by perilla seed extract belongs to mixed corrosion inhibitor, its adsorption behavior accords with Langmuir adsorption theory and its adsorption free energy is −22.70 kJ/mol.
(2) In the corrosion inhibitor solution, luteolin and apigenin are mainly adsorbed parallel to the surface of carbon steel to form a film. The adsorption mechanism is that carbonyl O atoms in luteolin and apigenin hybridize with the 3 d empty orbit of Fe.
(3) From the point of view of quantum chemistry, the smaller the HOMO value and the energy gap value, the better the adsorption of the corrosion inhibitor on the surface of carbon steel. From the point of view of molecular dynamics simulation, the greater the absolute value of adsorption energy, the better the adsorption of the corrosion inhibitor on carbon steel surface.

The follow-up research work can also test and evaluate the practical application of this new corrosion inhibitor and consider its comprehensive influence on concrete performance and reasonable compounding with other additives.

## Figures and Tables

**Table 1:** Analysis results of potentiodynamic polarization curves of carbon steel electrodes in different concentrations of corrosion inhibitor solutions.

Inhibitor Concentration (g/L)	E_corr_ (mV)	i_corr_ (A/cm^2^)	IE%
0	−931	1.11 × 10^−4^	-
1	−922	3.84 × 10^−5^	65.41
2	−900	3.16 × 10^−5^	71.49
3	−884	1.52 × 10^−5^	86.27
4	−875	1.18 × 10^−5^	89.39

**Table 2:** Chemical calculation results of corrosion inhibitor components.

Component	E_HOMO_ (eV)	E_LUMO_ (eV)	ΔE (eV)
luteolin	−5.045	−2.474	2.571
apigenin	−5.429	−2.779	2.650
α-linolenic acid	−5.505	−1.058	4.447

**Table 3:** Fukui function of luteolin.

Atom	f_i_^+^	f_i_^−^	Atom	f_i_^+^	f_i_^−^
C1	0.033	0.031	O7	0.033	0.037
C2	0.026	0.011	O8	0.024	0.015
C3	0.008	0.019	O9	0.027	0.034
C4	0.007	0.019	O13	0.092	0.280
C5	0.023	0.028	O20	0.028	0.026
C6	0.030	0.031	O21	0.052	0.041
C10	0.070	0.045	H22	0.023	0.025
C11	0.055	0.024	H23	0.016	0.022
C12	0.057	0.061	H24	0.015	0.018
C14	0.027	0.005	H25	0.020	0.026
C15	0.043	0.016	H26	0.026	0.022
C16	0.027	0.022	H27	0.022	0.010
C17	0.056	0.034	H28	0.026	0.019
C18	0.035	0.023	H29	0.021	0.009
C19	0.039	0.019	H30	0.016	0.012
			H31	0.021	0.016

**Table 4:** Fukui function of apigenin.

Atom	f_i_^+^	f_i_^−^	Atom	f_i_^+^	f_i_^−^
C1	0.032	0.053	O7	0.040	0.104
C2	0.031	0.036	O8	0.034	0.027
C3	0.024	0.104	O9	0.034	0.046
C4	0.009	0.026	O20	0.050	0.039
C5	0.012	0.037	O21	0.079	0.043
C6	0.025	0.046	H22	0.024	0.033
C10	0.058	0.017	H23	0.018	0.040
C11	0.051	0.056	H24	0.014	0.023
C12	0.074	0.029	H25	0.018	0.025
C14	0.024	0.006	H26	0.026	0.027
C15	0.041	0.018	H27	0.021	0.010
C16	0.034	0.024	H28	0.026	0.018
C17	0.056	0.031	H29	0.026	0.018
C18	0.035	0.026	H30	0.022	0.007

**Table 5:** Fukui function of α-linolenic acid.

Atom	f_i_^+^	f_i_^−^	Atom	f_i_^+^	f_i_^−^
O1	0.096	0.006	H26	0.008	0.000
C2	0.177	0.001	H27	0.008	0.000
O3	0.179	0.006	H28	0.007	0.005
C4	0.033	0.001	H29	0.007	0.005
C5	0.005	0.002	H30	0.002	0.000
C6	0.006	0.001	H31	0.002	0.000
C7	0.005	0.003	H32	0.005	0.010
C8	0.002	0.002	H33	0.005	0.010
C9	0.003	0.005	H34	0.001	0.012
C10	0.002	0.008	H35	0.001	0.012
C11	0.008	0.079	H36	0.007	0.037
C12	0.008	0.047	H37	0.008	0.027
C13	0.004	0.021	H38	0.002	0.036
C14	0.024	0.093	H39	0.003	0.036
C15	0.019	0.094	H40	0.014	0.042
C16	0.009	0.021	H41	0.013	0.042
C17	0.030	0.042	H42	0.012	0.036
C18	0.034	0.073	H43	0.012	0.035
C19	0.005	0.009	H44	0.016	0.025
C20	0.004	0.009	H45	0.017	0.034
H21	0.045	0.001	H46	0.005	0.014
H22	0.052	0.000	H47	0.005	0.013
H23	0.053	0.000	H48	0.006	0.012
H24	0.012	0.003	H49	0.006	0.012
H25	0.011	0.003	H50	0.003	0.015

**Table 6:** Adsorption energy of corrosion inhibitor molecules.

Adsorption Surface	Luteolin (kJ/mol)	Apigenin (kJ/mol)	α-Linolenic Acid (kJ/mol)
Fe (110)	−144.175	−141.949	−133.046
γ-FeOOH (010)	−33.293	−33.608	−27.304

## Data Availability

The data that support the findings of this study are available from the corresponding author upon reasonable request.
